# Morphology engineering of nickel molybdate hydrate nanoarray for electrocatalytic overall water splitting: from nanorod to nanosheet†

**DOI:** 10.1039/c8ra07323f

**Published:** 2018-10-12

**Authors:** Jianghao Wang, Liping Li, Lingshen Meng, Liping Wang, Yifeng Liu, Wenwen Li, Wengang Sun, Guangshe Li

**Affiliations:** Key Laboratory of Design and Assembly of Functional Nanostructures, Fujian Institute of Research on the Structure of Matter, Chinese Academy of Sciences Fuzhou 350002 P. R. China guangshe@jlu.edu.cn; University of Chinese Academy of Sciences Beijing 100049 P. R. China; State Key Laboratory of Inorganic Synthesis & Preparative Chemistry, College of Chemistry, Jilin University Changchun 130012 P. R. China

## Abstract

The morphology of nano-arrays plays an important role in their applications for catalysis, energy, environment. However, the morphology modulation of nano-arrays generally involves complex optimization of synthetic conditions including surfactants, pH, and solvent. In this work, we synthesize a NiMoO_4_·H_2_O nano-array by a simple hydrothermal method under mild conditions (pH = 6.47, aqueous solution, and without the aid of surfactants). The morphology modulation of the NiMoO_4_·H_2_O nano-array is realized by simply changing the hydrothermal temperature. When the hydrothermal temperature below 150 °C, a NiMoO_4_·H_2_O nanorod array is obtained. While the hydrothermal temperature is as high as 180 °C, the array on Ni foam is nanosheet instead of nanorod. The NiMoO_4_·H_2_O nanorod array synthesized at 150 °C shows a superior water splitting activity compared to the NiMoO_4_·H_2_O nanosheet array, affording a large current density of 10 mA cm^−2^ at an overpotential of <240 and 200 mV toward oxygen evolution reaction and hydrogen evolution reaction, respectively. Furthermore, the electrolyzer using NiMoO_4_·H_2_O nanorod array as both anode and cathode electrodes for catalyzing overall water splitting exhibits great performance, obtaining a current density of 10 mA cm^−2^ at 1.67 V, comparable to the integration of commercial noble-metal Pt/C and IrO_2_ electrodes.

## Abstract 2

We realize the modulation of nanoarray morphology from nanorod to nanosheet and improve the catalytic activity for water splitting by simply changing the hydrothermal temperature.

## Graphical abstract

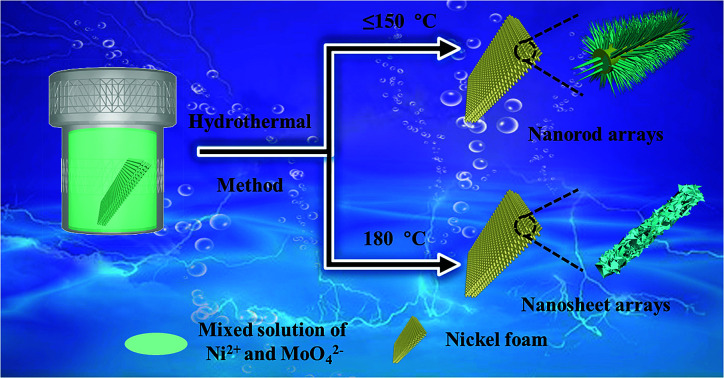

## Introduction

There is an emergent need for the development of new clean energy technologies, especially those based on water splitting, to meet the ever-increasing power demand in our daily life.^1,2^ Much attention is focused on the design of advanced electrocatalysts for water splitting. Some commercial noble metal-based nanomaterials, such as Pt/C, IrO_2_ and RuO_2_, exhibit low overpotential, small Tafel slope, and high exchange current density, but are still facing severe bottleneck problems including rare resources, high price, and poor electrochemical stability as well as their single performance.^3–6^ Hence, developing inexpensive and efficient catalysts as alternatives to Pt-based materials for hydrogen evolution reaction (HER) and IrO_2_ for oxygen evolution reaction (OER) is a crucial step for boosting widespread application of water splitting in the energy conversion process.^7,8^

Nanoarrays grown on a substrate exhibit great potential for water splitting due to the following advantages: (i) nanoarray can be directly used as a working electrode for water splitting without the aid of polymer binders, which is beneficial for exposing active sites promoting electrolyte penetration; (ii) nanoarray has a strong interaction with the conductive substrate, which facilitates electron transportation;^9,10^ (iii) nanoarray structure is propitious to the superaerophilic ability of electrode, and thus release H_2_ or O_2_ easier during water splitting.^11–13^ Fascinated by the merits of nanoarray, various nanoarrays, such as nanorod, nanosheet, and hierarchical structure have been synthesized successfully by precise chemical methods. But, it is still hard to realize the morphology change of nanoarray selectively by a simple optimization. The syntheses of these nanoarrays generally involve complicate optimizations of reaction conditions including surfactant, pH, and dissolvent, which damage their further application.^14–17^ Therefore, it is an emergency to develop a new simple method to modulate the morphology of nanoarray.

To this end, we take NiMoO_4_·H_2_O as an example, the modulation of nanoarray morphology from nanorod to nanosheet is realized by simply changing the hydrothermal temperature without the aids of surfactants or additives. We can obtain nanorod array when hydrothermal temperature below or equal to 150 °C. While the hydrothermal temperature is as high as 180 °C, the array on Ni foam is nanosheet instead of nanorod. In addition, the performance of NiMoO_4_·H_2_O nanorod array as bifunctional catalysts toward overall water splitting depends highly on the hydrothermal temperature in the sample preparation. When hydrothermal temperature equals to 150 °C, the obtained sample shows a superior OER and HER activity performance separately, affording a large current density of 10 mA cm^−2^ at an overpotential of <240 and 200 mV toward oxygen evolution reaction and hydrogen evolution reaction, respectively. As expected, the electrolyzer using NiMoO_4_·H_2_O nanorod array as both anode and cathode for catalyzing overall water splitting exhibited the great performance, obtaining a current density of 10 mA cm^−2^ at 1.67 V, comparable to the integration of commercial noble-metal Pt/C and IrO_2_ electrodes.

## Experimental section

### Materials

All reagents were purchased from commercial sources and used as received without further purification. Potassium hydroxide (KOH), Nickel nitrate hexahydrate (Ni (NO_3_)_2_·6H_2_O), sodium molybdate dihydrate (Na_2_MoO_4_·2H_2_O), Pt/C (20 wt%), IrO_2_ were purchased from Sinopharm Chemical Reagent Co. Ltd. (Beijing, China). All aqueous solution was prepared using ultrapure water (>18 MΩ).

### Synthesis of NiMoO_4_/NF

To remove the surface nickel oxide, Ni foam (2 cm × 3 cm) was ultrasonically cleaned by acetone and HCl solution (3 M) for 15 min in turn and subsequently rinsed with water and ethanol for three times. The cleaned Ni foam was immersed into 60 mL of a solution that contains 3 mmol Ni(NO_3_)_2_·6H_2_O and 3 mmol Na_2_MoO_4_·2H_2_O in a Teflon-lined stainless autoclave (100 mL). The solution pH at which hydrothermal treatment is about 6.47. Then the autoclave was sealed and treated in an oven at 150 °C for 6 h. The resultant sample was washed with ethanol three times and finally dried at 60 °C for 2 h. To study the effect of temperature on the formation of NiMoO_4_ nanorod array, the similar processes were performed in different reaction temperature (120 °C, 180 °C). For convenience, the products were marked as NMO-*T* (*T* is the hydrothermal temperature).

To investigate the growth mechanisms of nickel molybdate hydrate nanoarray, we did some control experiments including the Ni-salt precursor, hydrothermal time, conductivity substrate, and concentration of Ni-salt precursor. Detailed experimental conditions are shown in Table S1.†

### Characterization and instrumentations

Phase structures of the samples were characterized by powder X-ray diffraction (XRD) (Cu Kα, *λ* = 1.5418 Å) on a Rigaku Miniflex apparatus. Raman spectrum was recorded for powder sample in the backward geometry on Labram HR800 Evolution using the excitation wavelength of 532 nm from a He–Ne laser at room temperature. Scanning electron microscopy (SEM) performed on JSM-6700F and transmission electron microscopy (TEM) on F20 were used to study the morphologies of the samples. X-ray photoelectron spectroscopy (XPS) measurements were achieved using an ESCALAB 250Xi spectrometer (Thermo Fischer Scientific) equipped with an Al Kα monochromatic source. The charging shift was calibrated the C 1s photoemission line at a binding energy of 284.8 eV.

### Electrochemical characterization

All electrochemical measurements were carried out on a CHI 760E electrochemical analyzer (CHI Instruments, Inc., Shanghai) in a standard three-electrode system. In this work, the potentials were displayed *versus* RHE by the RHE calibration: *E* (RHE) = *E* (Hg/HgO) + 0.098 + 0.0591 × pH. Linear scan voltammetry (LSV) was measured with a scan rate of 5 mV s^−1^ and all results were revised by the ohmic potential drop (*iR*) correction. Cyclic voltammetry (CV) measurements with different scan rates (40, 80, 120, 160, 200 mV s^−1^) were used to determine the electrochemical double layer capacitances (EDLC, *C*_dl_). The electrochemical impedance spectroscopy (EIS) measurements were carried out at an overpotential of 370 mV in the frequency range of 10^−2^ to 10^5^ Hz. Prior to all the above measurements, oxygen was bubbled into the electrolyte for 30 min.

For the comparative purpose, the different control materials in powdered forms (Pt/C, IrO_2_) were drop-casted on Ni foam. Their amount and working surface area were kept the same as those used for NMO-150. In typical procedure: (1) 7 mg of catalyst was dispersed in 1 mL mixture of 450 μL de-ionized water, 500 μL ethanol and 50 μL 5% Nafion to form a homogeneous mixture, (2) 500 μL of this solution was drop-casted onto Ni foam electrode with an area of 1 cm^2^ and a loading amount of 3.5 mg cm^−2^, and left to dry in air.

## Results and discussions

**Fig. 1:**
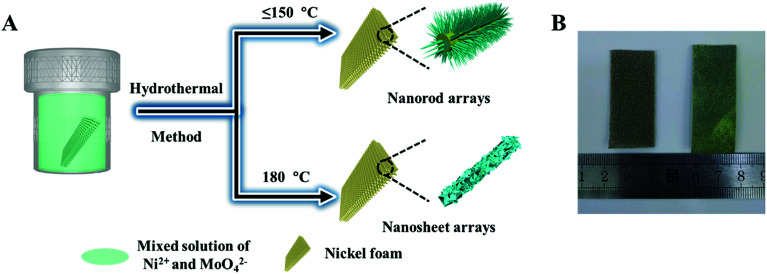
(A) Schematic illustration of the preparation process for NiMoO_4_ hydrate array and (B) photography of the bare Ni foam (left) and NMO-150 (right).

The NiMoO_4_·H_2_O nanorods and nanosheets were directly grown on commercial Ni foam by a hydrothermal method, as schematically elucidated in Fig. 1A. The cleaned Ni foam was immersed into a mixed solution of nickel nitrate and sodium molybdate (60 mL) in a Teflon-lined stainless autoclave. Then the autoclave was sealed and reacted at a selected temperature for 6 h. Fig. 1B illustrates optical pictures of the primitive Ni foam and sample NMO-150. The color of Ni foam is changed from silver to yellow after the hydrothermal reaction, indicating that the active materials are uniformly coated on the Ni foam.

**Fig. 2:**
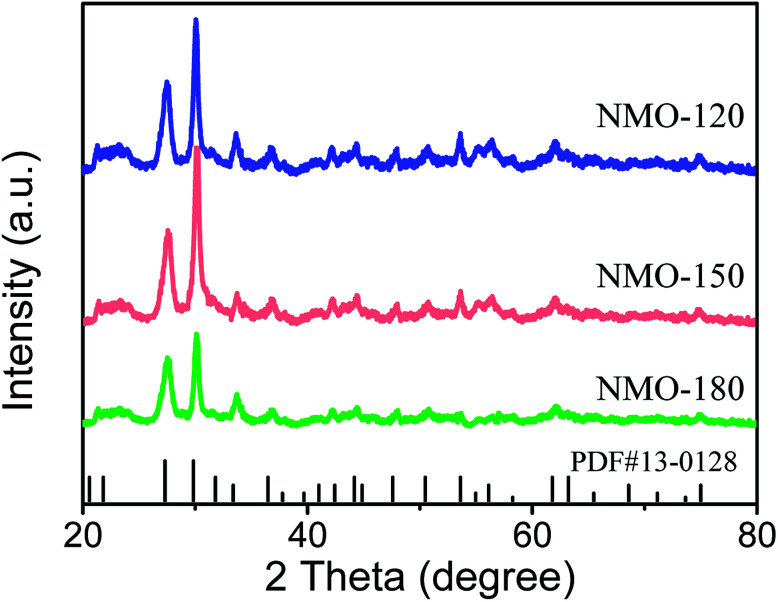
XRD patterns for NMO-120, NMO-150, NMO-180. The data were recorded for the powder that was scraped from Ni foam.

To optimize the synthetic temperature of the growth of nanoarray, we adjusted the reaction temperature from 120 °C to 180 °C. The phase structures of NiMoO_4_·H_2_O nanoarrays were characterized by X-ray diffraction (XRD, Fig. 2). As shown in Fig. 2, the nanoarrays prepared under 120, 150, and 180 °C have a similar XRD pattern. In addition, the diffraction peak of XRD pattern in Fig. 2 agrees well with the data for NiMoO_4_·H_2_O (JCPDS no. 13-0128), revealing that all nanoarrays grown on Ni substrate are NiMoO_4_·H_2_O.^18^ In addition, Raman spectroscopy is used to monitor the structure of NMO-150. As shown in Fig. S1,† a set of Raman peaks are observed at 805, 831, and 938 cm^−1^, which are in consistent with previously reported Mo–O vibrations of NiMoO_4_.^19^ The Raman spectrum also demonstrates the existence of NiMoO_4_·H_2_O in the sample.

**Fig. 3:**
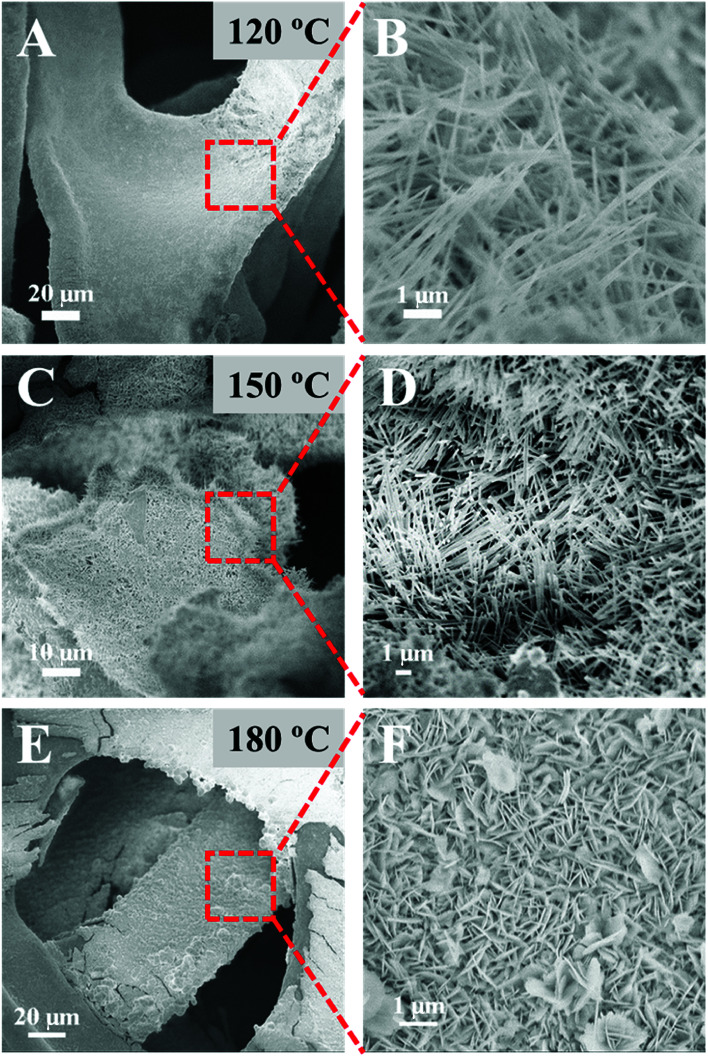
Low-magnification (left panel) and high-magnification (right panel) SEM images of NMO-*T* samples synthesized at the hydrothermal temperature of 120 °C (A and B), 150 °C (C and D) and 180 °C (E and F).

Fig. 3 shows the scanning electron microscopy (SEM) images of NMO-120, NMO-150, and NMO-180. The low-magnification SEM images in the left panel (Fig. 3(A, C, and E)) reveal that all samples are covered by a rough shell, indicating the successful growth of NiMoO_4_·H_2_O nanoarrays. The high-magnification SEM images shown in Fig. 2(B and D) demonstrate that the nanorods are covered on Ni foam for the samples that were obtained at the hydrothermal temperature of 120 °C or 150 °C. Comparatively, when the hydrothermal temperature is as high as 180 °C, the array on Ni foam is nanosheet instead of nanorod (Fig. 2F). This interesting finding demonstrates that hydrothermal temperature is an important parameter for the growth of nanoarrays. Different morphology of nanoarray from nanorod to nanosheet can be obtained by simply changing the hydrothermal temperature without the aid of surfactants or additives. The effect of temperature on the growth of nanoarrays is changing the Ni source that reacts with MoO_4_^2−^ ions from Ni^2+^ ions to Ni foam. When the reaction temperature below or equal to 150 °C, MoO_4_^2−^ ions react with Ni^2+^ ions in the solution, and then NiMoO_4_·H_2_O nanorods grow on the Ni foam directly. While reaction temperature equal to 180 °C, MoO_4_^2−^ ions react with Ni foam directly to form nanosheet array,^20,21^ which impedes the growth of NiMoO_4_·H_2_O nanorod. To verify this suppose, some control experiments were performed (Table S1†). Firstly, we changed the Ni-precursor by NiCl_2_·6H_2_O and NiSO_4_·6H_2_O. From Fig. S2,† we find that nanorod array can still be obtained under similar reaction conditions when using NiCl_2_·6H_2_O and NiSO_4_·6H_2_O as Ni-salt precursor, which demonstrate that the change in Ni-precursor has little effect on the morphological evolution. Secondly, we have replaced Ni foam with carbon cloth under the 180 °C for 6 h. As shown in Fig. S3,† we find that nanorod instead of nanosheet array can be obtained, providing the evidence of that the Ni^2+^ ions of NMO-180 are origin from Ni foam. Thirdly, we have changed the concentrations of Ni-salt (0, 6 mmol) to investigate the contribution of Ni^2+^ ions. From Fig. S4,† one can find that when the concentration of Ni-salt was 0 mmol, only the nanosheet array can be obtained, indicating that when Ni^2+^ ions come from the Ni foam, the dimensional anisotropy would be changed from 1D to 2D. When the concentration of Ni-salt was 6 mmol, only the nanorod array can be obtained, suggesting that additional contribution of Ni^2+^ ions would not result in the sheet-like structure.

NMO-120, NMO-150, NMO-180 were prepared under different hydrothermal temperature, which might result in a different activity for water splitting. To verify this suppose, the catalytic tests of oxygen evolution reaction (OER) and hydrogen evolution reaction (HER) for these three samples were performed in 1 M KOH electrolyte (Fig. S5 and S6†). As shown in (Fig. S5 and S6†), the sample of NMO-150 exhibits superior catalytic performance both in the OER and HER processes than other samples. This is because of the following two reasons. On the one side, the thickness of NiMoO_4_ films over Ni foam varied with hydrothermal temperature. From Fig. S5,† we can find that the oxidation peak of Ni^2+^/Ni^3+^ become weak with the increase of hydrothermal temperature, suggesting that the thickness of NiMoO_4_ films also become thinner. The nanosheet grown on the Ni foam would impede the growth of NiMoO_4_·H_2_O nanorod and decrease the amount of active species on the Ni substrate. Therefore, NMO-180 may have a less activity than NMO-150. On the other side, although nanorods covered Ni foam were prepared at both 120 °C and 150 °C, higher temperature (150 °C) could enhance the synergistic effect between NiMoO_4_ and Ni foam, which is beneficial to catalysis. Hence, the reaction temperature of 150 °C is the optimum condition for the growth of NiMoO_4_·H_2_O nanorod array, and thus NMO-150 is picked to systemically study its physical properties and catalytic performances.

**Fig. 4:**
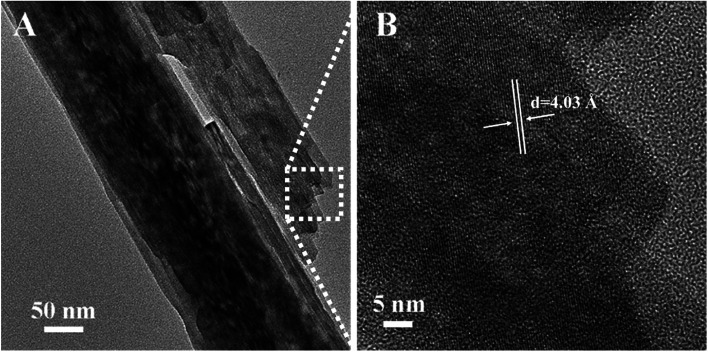
TEM (A) and HRTEM (B) of NMO-150. The powder for the TEM was scraped from Ni foam.

To study the effect of hydrothermal time on the growth of nanorod array, we controlled the hydrothermal time (2 h, 4 h) under similar condition. From Fig. S7,† we can find that the samples treated by the hydrothermal method for 2 h and 4 h have a rod-like structure. With the increase of hydrothermal time, the length of nanorod become longer, which demonstrate that the growth mechanism of nickel molybdate hydrate nanoarray obeys the orientation attachment growth. Transmission electronic microscope (TEM) was performed to further reveal the morphology of NMO-150. As shown in Fig. 4A, the nanorod with a size about 150 nm in diameter can be clearly seen, which is consistent with the observation of SEM. Moreover, a large number of mesopores can be found easily on the surface of the nanorod. Measurements of isothermal (77 K) N_2_ adsorption and desorption for NMO-150 were performed to characterize the surface area and porosity of the product (Fig. S8†). The average pore diameter of the NMO-150 is about 3–5 nm to the desorption data (Fig. S8†). The existence of the mesoporous structure can not only increase the surface area of the catalyst but also provide more defects to produce an excellent catalytic performance. The corresponding high-resolution TEM (Fig. 4B) shows that the spacing between two adjacent lattice fringes is 4.03 Å.

**Fig. 5:**
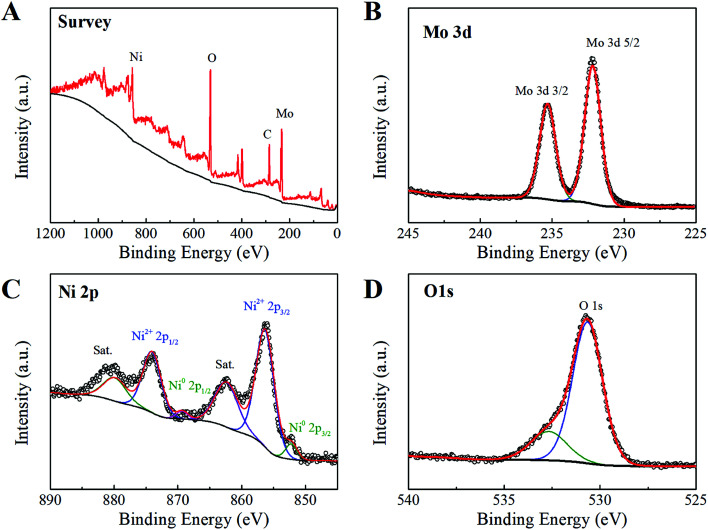
X-ray photoelectron spectrum of NiMoO_4_·H_2_O: (A) survey spectrum, (B) Mo 3d, (C) Ni 2p and (D) O 1s.

The detailed surface chemical feature of NMO-150 was further determined by X-ray photoelectron spectroscopy (XPS). The XPS survey spectrum (Fig. 5A) shows typical signals of Ni, Mo, O elements, consistent with EDS analysis (Fig. S9†). In the Mo 3d core-level spectrum (Fig. 5A), two peaks at 232.2 and 235.3 eV are attributed to Mo 3d_5/2_ and Mo 3d_3/2_ respectively, and the spin-orbital splitting is about 3.0 eV (ΔMo 3d). The values for the binding energy of Mo 3d_5/2_ and ΔMo 3d are the typical features of the Mo^6+^ ion,^22^ which suggests that valence state of Mo ions in NMO-150 is +6. The Ni 3p in Fig. 5C exhibits a complicate spectrum comparing to pure Ni^2+^ and metal nickel.^21^ In addition to the strong photoelectron peaks at 856.3 and 874.2 eV and two satellite peaks at high binding energy side, two peaks at low binding energy are also distinguished. Therefore, the Ni 2p spectrum was de-convoluted into two spin–orbit doublets. The strong doublet at binding energies of 856.3 and 874.2 eV is ascribed to Ni^2+^, while the other one at 852.3 and 869.3 eV is assigned to Ni^0^ that comes from Ni foam.^23^ The O 1s signals with binding energies of 530.7 eV and 532.7 eV correspond to oxygen species in lattice and hydrate, respectively.^3,24^ According to the above XPS results, it can be concluded that the valences of Ni, Mo, and O elements are +2, +6, and −2, respectively. These observations indicate that NiMoO_4_ hydrate nanorod array has been synthesized successfully through a convenient hydrothermal method.

**Fig. 6:**
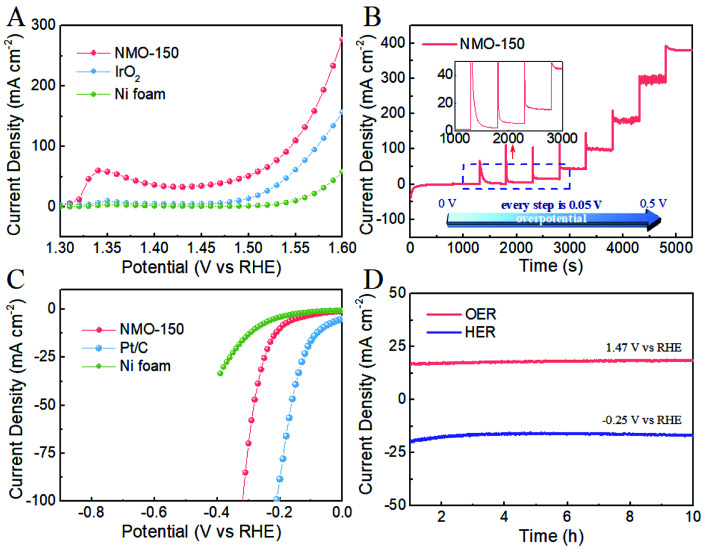
(A) and (C) LSV curves of NMO-150, IrO_2_, Ni foam for OER and HER in 1 M KOH solution with iR correction at a sweep rate of 5 mV s^−1^. (B) The multi-step chronoamperometric curve obtained with NMO-150 in 1 M KOH solution, measured at different overpotentials, starting at 1.23 V and ending at 1.73 V (*vs.* RHE) with an increment of 50 mV every 500 s. (D) Current density *versus* time (*I*–*t*) curve obtained over NMO-150 at 1.47 V and −0.25 V (*vs.* RHE) over 10 h long electrocatalytic OER and HER.

We evaluated the electrocatalytic activity of NMO-150 for OER and HER in 1 M KOH solution by a typical three-electrode system, in which NMO-150 was tailored into 1 × 1 cm^2^ and directly used as the working electrode. For comparison, we also measured the electrocatalytic activity of Ni foam, Ni foam-supported of commercial IrO_2_. As shown in Fig. 6A, NMO-150 shows a remarkable catalytic activity toward OER, with a current density of ≈280 mA cm^−2^ at a small overpotential of 370 mV, which is superior to many electrocatalysts reported previously.^20,25–28^ Moreover, the activity of NMO-150 is apparently better than that of IrO_2_, the benchmark for OER. Specifically, NMO-150 achieves a current density of 100, 150, 200 mA cm^−2^ with small overpotentials of about 310, 330, 350 mV, respectively. Where to reach the current density of 100 and 150 mA cm^−2^, the IrO_2_ must be applied larger overpotentials of 340 and 370 mV, respectively. Furthermore, at an overpotential of 370 mV, the current density of NMO-150 is about two and six times larger than commercial IrO_2_ and Ni foam. These results confirm the excellent OER performance of NMO-150.

Fig. 6B shows a multi-step chronoamperometric curve of NMO-150 toward OER in 1 M KOH solution. The operated overpotential was applied starting at 1.23 V and ending at 1.73 V (*vs.* RHE) with an increment of 50 mV every 500 s. The current maintains stable at each potential in the whole range, from 50 to 550 mV, and the current also switch quite rapidly, suggesting that NMO-150 has an excellent stability in a wide current density range (0–400 mA cm^−2^) and good mass transport property.^29^

We also examined the catalytic property of NMO-150 toward HER in basic media. For comparison, the electrocatalytic activity of Ni foam and Ni foam-supported of commercial Pt/C (20 wt%) were measured under the same testing condition. As shown in Fig. 6C, although the activity of NMO-150 is lower than that of Pt/C (20 wt%), much higher than that of Ni foam. When overpotential is 0.3 V, the current density for NMO-150 is about 75 mA cm^−2^, which is nearly seven times larger than Ni foam. To evaluate the stability of NMO-150, current density–time (*I*–*t*) curves for OER and HER in 1 M KOH were recorded at the potential of 1.47 V and −0.25 V (*vs.* RHE). As shown in Fig. 6D, NMO-150 retains the current density at 15 mA cm^−2^ over as long as 10 h electrocatalytic OER and HER. This further demonstrates that NMO-150 is a highly stable electrocatalyst for both OER and HER in basic media.

**Fig. 7:**
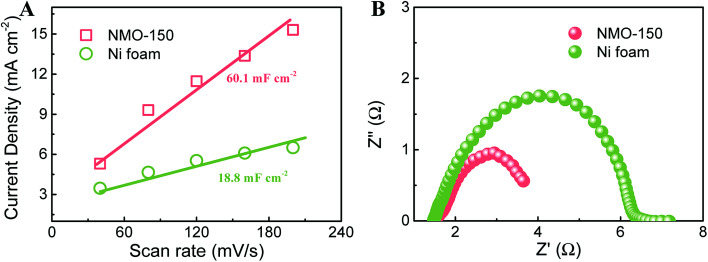
(A) Relationship between current density and scan rate, and (B) EIS Nyquist plots recorded at an overpotential of 270 mV for NMO-150 and Ni foam.

The electrochemical double layer capacitances (EDLC) were measured to evaluate the active area of electrocatalysts. Fig. 7A compares the variation of current density with scan rate for NMO-150 and Ni foam. Their double-layer capacitance (*C*_dl_) values can be calculated by the slopes.^3,30,31^ The value of *C*_dl_ for NMO-150 is 60.1 mF cm^−2^, about three times larger than Ni foam, indicating that NMO-150 possesses a larger active area among all samples studied. Electrochemical impedance spectroscopy (EIS) for NMO-150 and Ni foam was recorded in 1 M KOH solution at an overpotential of 270 mV to determine their electrical conductivity. The equivalent circuit is shown in Fig. S10.† As illustrated in Fig. 7B and Table 1, comparing to Ni foam, sample NMO-150 exhibits smaller *R*_ct,2_ (charge transfer resistance) and. The smaller *R*_ct,2_ suggests that the charge transfer in the NMO-150 is more fluent, which could give a higher reaction rate.^32–34^ Therefore, the excellent catalytic activity of NMO-150 can attribute to a large number of active sites and improved electrical conductivity.

**Table 1:** Resistances and capacities derived from EIS fitting according to the circuit shown in Fig. 7C

Samples	*R*_s_ (Ω)	*R*_ct,1_ (Ω)	CPE_1_-p	*R*_ct,2_ (Ω)	CPE_2_-p
NMO-150	1.48	0.55	0.44	1.89	0.94
Ni foam	1.36	0.19	0.77	3.03	0.72

**Fig. 8:**
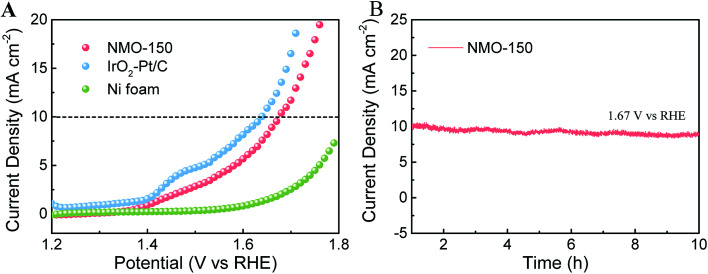
(A) Comparison of LSV curves detected for three electrolyzers containing NMO-150-NMO-150, Pt/C–IrO_2_ and Ni foam-Ni foam couple, respectively, for overall water splitting with *iR* correction at a scan rate of 5 mV s^−1^. (B) The chronoamperometric curve of water electrolysis for NMO-150 in a two-electrode configuration at a static potential of 1.67 V.

Given our finding that NMO-150 has an exotic bi-functional property toward OER and HER, we assembled an electrolyzer with NMO-150 as anode and cathode to study its activity for overall water splitting (OWS). As illustrated in Fig. 8A, the electrolyzer using NMO-150 as anode and cathode affords current density of 10 mA^−2^ at 1.67 V (*vs.* RHE). Despite this current density value is slightly inferior to that of electrolyzer constructed by Pt/C–IrO_2_ couple, it outperforms state-of-the-art electrolyzers utilizing Co- or Ni-based bifunctional electrocatalysts for overall water splitting.^34^ Furthermore, the long-term stability of this system was also tested. When the static potential of 1.67 V was applied, the electrolyzer afforded a constant current density around 10 mA cm^−2^ and showed an excellent stability after testing for 10 h (Fig. 8B).

## Conclusion

In summary, NiMoO_4_·H_2_O nanorod and nanosheet arrays are prepared by a simple one-pot hydrothermal method. We can realize the morphological change of array from nanorod to nanosheet by only tuning the hydrothermal temperature without the aids of surfactants or additives. Nanorod array can be obtained when hydrothermal temperature below or equal to 150 °C. While the hydrothermal temperature is as high as 180 °C, the array on Ni foam is nanosheet instead of nanorod. Electrochemical tests showed that the nanorod array synthesized at 150 °C possesses remarkable catalytic activity toward both OER and HER. NiMoO_4_·H_2_O nanorod array afforded current density of 10 mA cm^−2^ for OER and HER at small overpotentials of <240 mV and 200 mV, respectively. Furthermore, the electrolyzer with NiMoO_4_·H_2_O nanorod array as electrocatalyst at both sides of the electrodes also exhibited high performance for the overall water splitting reaction. In consideration of its excellent catalytic activity and the facile and scalable synthesis, NiMoO_4_ hydrate nanorod array might hold great promise as a substitutable non-noble metal-based electrode material towards large-scale electrical water splitting.

## Conflicts of interest

The authors declare no competing financial interest.

## Supplementary Material

RA-008-C8RA07323F-s001

## References

[cit1] Seh Z. W., Kibsgaard J., Dickens C. F., Chorkendorff I., Nørskov J. K., Jaramillo T. F. (2017). Science.

[cit2] Zhu Y., Ji X., Yin R., Hu Z., Qiu X., Wu Z., Liu Y. (2017). RSC Adv..

[cit3] Wang J., Li L., Tian H., Zhang Y., Che X., Li G. (2017). ACS Appl. Mater. Interfaces.

[cit4] Yu M. Q., Jiang L. X., Yang H. G. (2015). Chem. Commun..

[cit5] Zhang B., Zheng X., Voznyy O., Comin R., Bajdich M., García-Melchor M., Han L., Xu J., Liu M., Zheng L., García de Arquer F. P., Dinh C. T., Fan F., Yuan M., Yassitepe E., Chen N., Regier T., Liu P., Li Y., De Luna P., Janmohamed A., Xin H. L., Yang H., Vojvodic A., Sargent E. H. (2016). Science.

[cit6] Liu T., Li M., Jiao C., Hassan M., Bo X., Zhou M., Wang H.-L. (2017). J. Mater. Chem. A.

[cit7] Zhu K., Wu T., Zhu Y., Li X., Li M., Lu R., Wang J., Zhu X., Yang W. (2017). ACS Energy Lett..

[cit8] Lin H., Li H., Li Y., Liu J., Wang X., Wang L. (2017). J. Mater. Chem. A.

[cit9] Chen Y.-Y., Zhang Y., Zhang X., Tang T., Luo H., Niu S., Dai Z.-H., Wan L.-J., Hu J.-S. (2017). Adv. Mater..

[cit10] Kuang Y., Feng G., Li P., Bi Y., Li Y., Sun X. (2016). Angew. Chem., Int. Ed..

[cit11] Wang J., Li L., Wang L., Liu Y., Sun W., Li W., Li G. (2018). ACS Omega.

[cit12] Lu Z., Sun M., Xu T., Li Y., Xu W., Chang Z., Ding Y., Sun X., Jiang L. (2015). Adv. Mater..

[cit13] Lu Z., Xu W., Ma J., Li Y., Sun X., Jiang L. (2016). Adv. Mater..

[cit14] Tang C., Cheng N., Pu Z., Xing W., Sun X. (2015). Angew. Chem., Int. Ed..

[cit15] Zhao Q., Zhong D., Liu L., Li D., Hao G., Li J. (2017). J. Mater. Chem. A.

[cit16] Tang C., Zhang R., Lu W., Wang Z., Liu D., Hao S., Du G., Asiri A. M., Sun X. (2017). Angew. Chem..

[cit17] Zhang J., Wang T., Liu P., Liao Z., Liu S., Zhuang X., Chen M., Zschech E., Feng X. (2017). Nat. Commun..

[cit18] Guo D., Luo Y., Yu X., Li Q., Wang T. (2014). Nano Energy.

[cit19] Wu Y., Li G.-D., Liu Y., Yang L., Lian X., Asefa T., Zou X. (2016). Adv. Funct. Mater..

[cit20] Rao Y., Wang Y., Ning H., Li P., Wu M. (2016). ACS Appl. Mater. Interfaces.

[cit21] Feng L.-L., Yu G., Wu Y., Li G.-D., Li H., Sun Y., Asefa T., Chen W., Zou X. (2015). J. Am. Chem. Soc..

[cit22] Zhang Z., Liu Y., Huang Z., Ren L., Qi X., Wei X., Zhong J. (2015). Phys. Chem. Chem. Phys..

[cit23] Yang Y., Zhang K., Lin H., Li X., Chan H. C., Yang L., Gao Q. (2017). ACS Catal..

[cit24] Chen S., Li L., Hu W., Huang X., Li Q., Xu Y., Zuo Y., Li G. (2015). ACS Appl. Mater. Interfaces.

[cit25] Jin Y., Wang H., Li J., Yue X., Han Y., Shen P. K., Cui Y. (2016). Adv. Mater..

[cit26] Liang J., Wang Y.-Z., Wang C.-C., Lu S.-Y. (2016). J. Mater. Chem. A.

[cit27] Meng J., Fu J., Yang X., Wei M., Liang S., Zang H.-Y., Tan H., Wang Y., Li Y. (2017). Inorg. Chem. Front..

[cit28] Pi Y., Shao Q., Wang P., Lv F., Guo S., Guo J., Huang X. (2017). Angew. Chem., Int. Ed..

[cit29] Wu Y., Liu Y., Li G.-D., Zou X., Lian X., Wang D., Sun L., Asefa T., Zou X. (2017). Nano Energy.

[cit30] Zhang J., Wang T., Pohl D., Rellinghaus B., Dong R., Liu S., Zhuang X., Feng X. (2016). Angew. Chem., Int. Ed..

[cit31] Fang L., Li W., Guan Y., Feng Y., Zhang H., Wang S., Wang Y. (2017). Adv. Funct. Mater..

[cit32] Wan S., Qi J., Zhang W., Wang W., Zhang S., Liu K., Zheng H., Sun J., Wang S., Cao R. (2017). Adv. Mater..

[cit33] Li J., Xu W., Zhou D., Luo J., Zhang D., Xu P., Wei L., Yuan D. (2018). J. Mater. Sci..

[cit34] Zhao X., Shang X., Quan Y., Dong B., Han G.-Q., Li X., Liu Y.-R., Chen Q., Chai Y.-M., Liu C.-G. (2017). Electrochim. Acta.

