# Supplementary material for: Morphology engineering of nickel molybdate hydrate nanoarray for electrocatalytic overall water splitting: from nanorod to nanosheet
Source: RSC Adv. 2018 Oct 12;8(61):35131–8. doi: 10.1039/c8ra07323f (PMC9087359; doi:10.1039/c8ra07323f)
Supplement: RA-008-C8RA07323F-s001 [file RA-008-C8RA07323F-s001.pdf]

Electronic Supplementary Material (ESI) for RSC Advances.
This journal is © The Royal Society of Chemistry 2018

Supporting Information

Morphology Engineering of Nickel Molybdate Hydrate Nanoarray for Electrocatalytic Overall Water Splitting: from Nanorod to Nanosheet

*Jianghao Wang,[1,2] Liping Li,[3] Lingshen Meng,[3] Liping Wang,[3] Yifeng Liu,[3] Wenwen Li,[3] Wengang Sun,[3] Guangshe Li[1,3]**

1. Key Laboratory of Design and Assembly of Functional Nanostructures, Fujian Institute of Research on the Structure of Matter, Chinese Academy of Sciences, Fuzhou 350002, P.R. China

2. University of Chinese Academy of Sciences, Beijing 100049, P.R. China

3. State Key Laboratory of Inorganic Synthesis & Preparative Chemistry, College of Chemistry, Jilin University, Changchun 130012, P.R. China

AUTHOR INFORMATION

Corresponding Author

* E-mail: guangshe@jlu.edu.cn

Table S1. Detailed condition of the control experiments for investigating the growth mechanism of nickel molybdate hydrate nanoarray

Control Experiment Number	Ni-salt Precursor	Mo-salt Precursor	Hydrothermal Temperature	Hydrothermal Time	Substrate
1	$Ni(NO_3)_2 \cdot 6H_2O$ 3 mmol	$Na_2MoO_4 \cdot 2H_2O$ 3 mmol	150 °C	2 h	Ni foam
2	$Ni(NO_3)_2 \cdot 6H_2O$ 3 mmol	$Na_2MoO_4 \cdot 2H_2O$ 3 mmol	150 °C	4 h	Ni foam
3	$Ni(NO_3)_2 \cdot 6H_2O$ 3 mmol	$Na_2MoO_4 \cdot 2H_2O$ 3 mmol	150 °C	6 h	Carbon Cloth
4	$Ni(NO_3)_2 \cdot 6H_2O$ 0 mmol	$Na_2MoO_4 \cdot 2H_2O$ 3 mmol	150 °C	6 h	Ni foam
5	$Ni(NO_3)_2 \cdot 6H_2O$ 6 mmol	$Na_2MoO_4 \cdot 2H_2O$ 3 mmol	150 °C	6 h	Ni foam
6	$NiCl_2 \cdot 6H_2O$ 3 mmol	$Na_2MoO_4 \cdot 2H_2O$ 3 mmol	150 °C	6 h	Ni foam
7	$NiSO_4 \cdot 6H_2O$ 3 mmol	$Na_2MoO_4 \cdot 2H_2O$ 3 mmol	150 °C	6 h	Ni foam

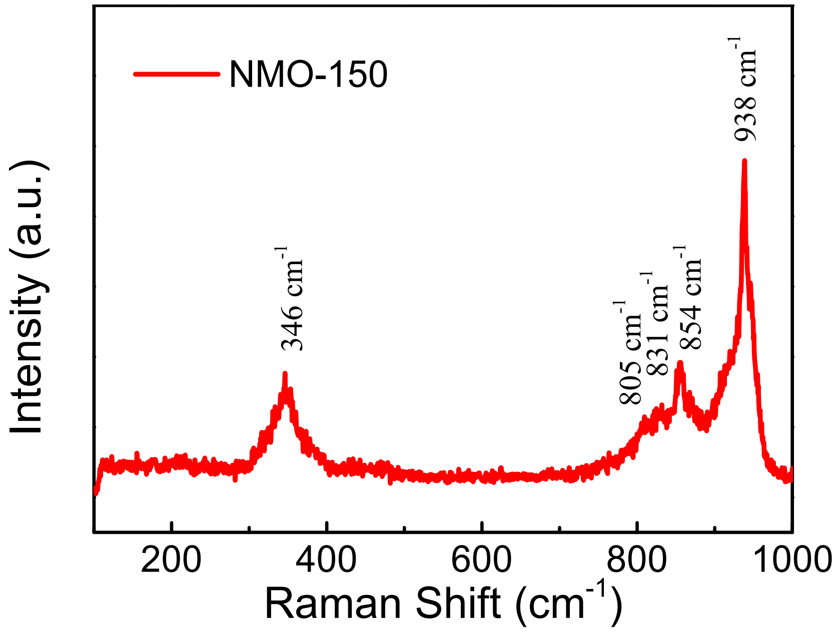

Figure S1. Raman spectrum of NMO-150. The data was recorded for the powder that was scraped from Ni foam.

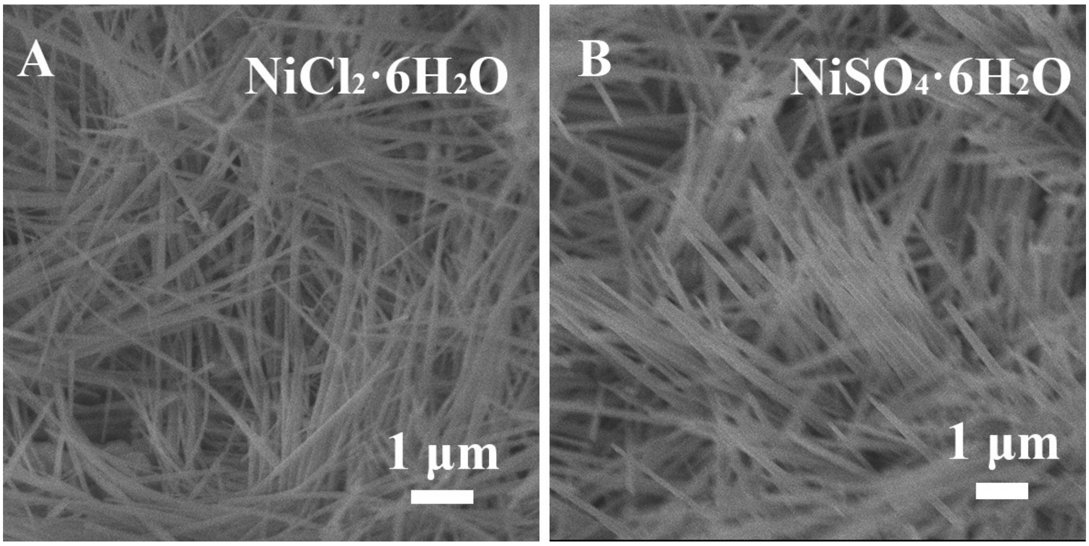

Figure S2. SEM images of NMO-150 samples synthesized with different Ni-salt precursors: (A) $NiCl_2 \cdot 6H_2O$; (B) $NiSO_4 \cdot 6H_2O$.

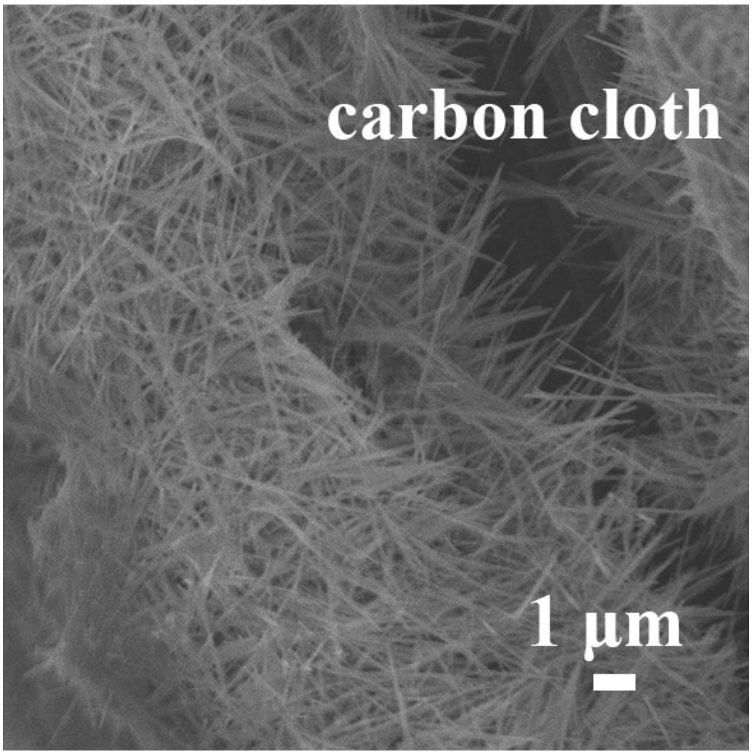

Figure S3. SEM image of nickel molybdate hydrate nanoarray grown on the carbon cloth.

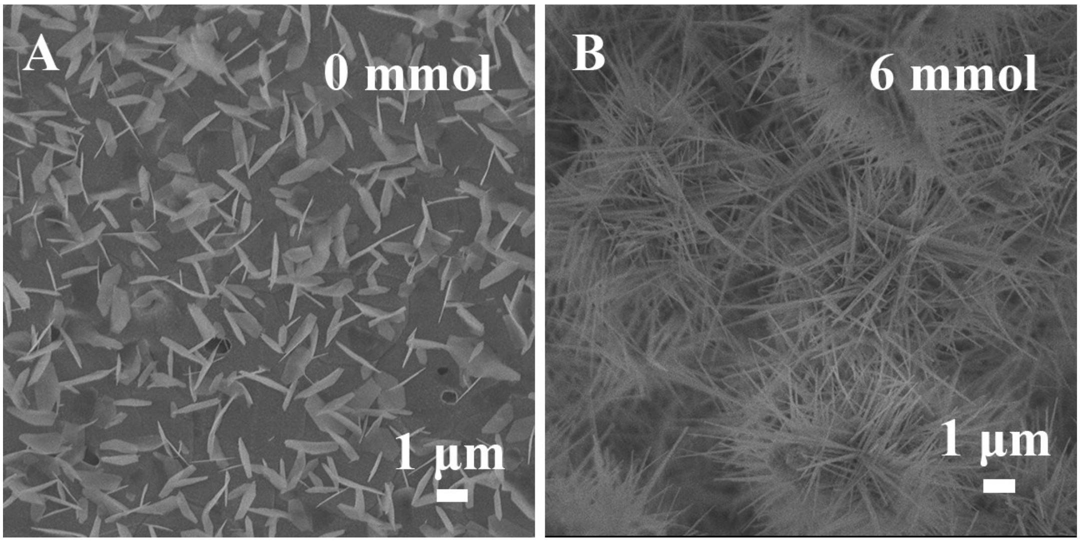

Figure S4. SEM images of nickel molybdate hydrate nanoarray synthesized with different concentration of Ni-salt precursor: (A) 0 mmol $Ni(NO_3)_2 \cdot 6H_2O$; (B) 6 mmol $Ni(NO_3)_2 \cdot 6H_2O$.

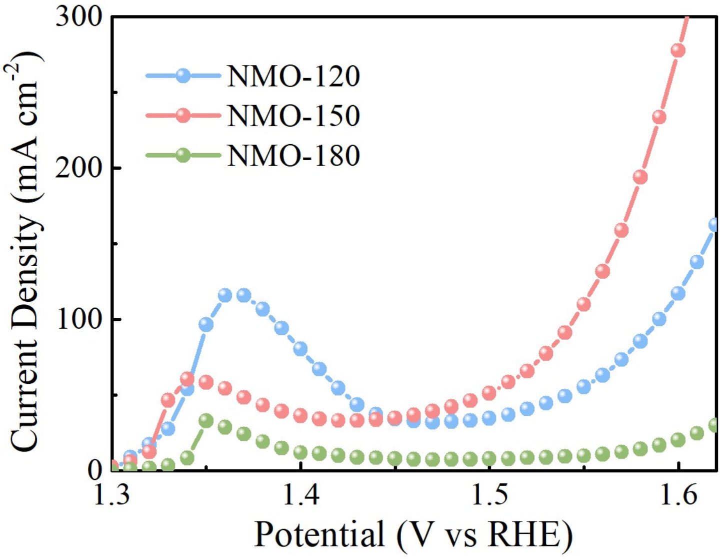

Figure S5. LSV curves of NMO-120, NMO-150, NMO-180 for OER in 1 M KOH solution with iR correction at a sweep rate of 5 mV/s.

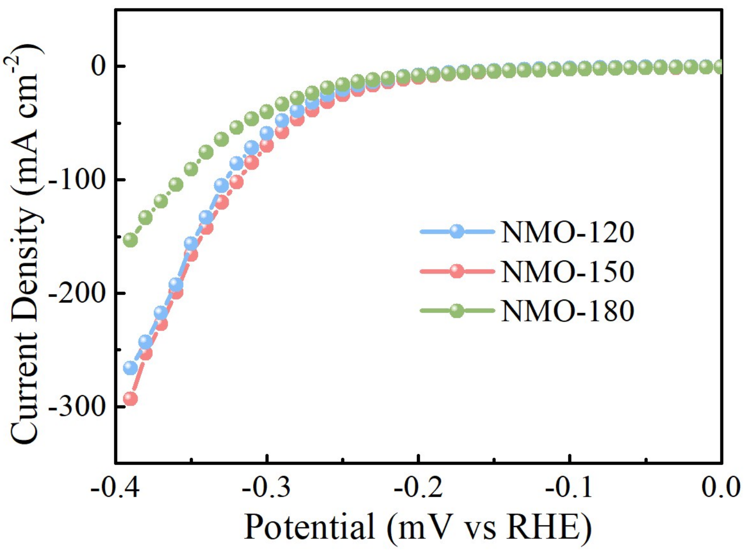

Figure S6. LSV curves of NMO-120, NMO-150, NMO-180 for HER in 1 M KOH solution with iR correction at a sweep rate of 5 mV/s.

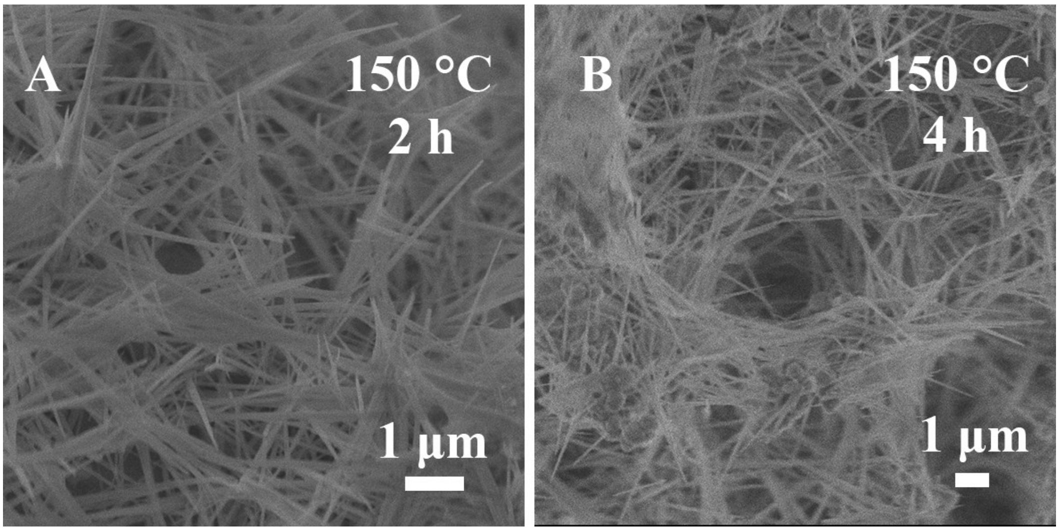

Figure S7. SEM images of nickel molybdate hydrate nanoarray synthesized at 150 °C for different hydrothermal time: (A) 2h; (B) 4h.

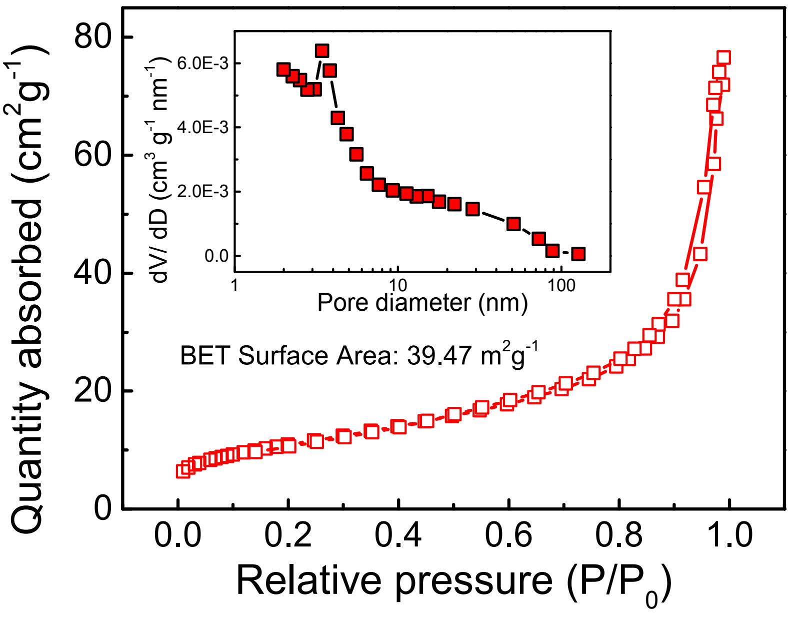

Figure S8. Nitrogen adsorption-desorption isotherms for NMO-150. Inset in Figure S8 is the corresponding pore size distribution.

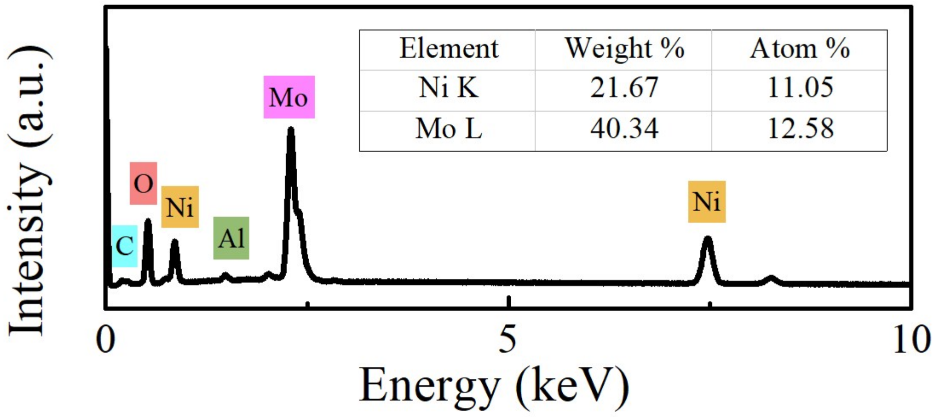

Figure S9. Energy dispersive spectrum of NMO-150

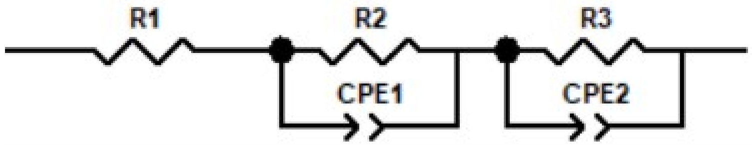

Figure S10. Equivalent electric circuit of the cell.
